# Black American women’s attitudes toward seeking mental health services and use of mobile technology to support the management of anxiety

**DOI:** 10.1093/jamiaopen/ooad088

**Published:** 2023-10-17

**Authors:** Terika McCall, Meagan Foster, Holly R Tomlin, Todd A Schwartz

**Affiliations:** Division of Health Informatics, Department of Biostatistics, Yale School of Public Health, New Haven, CT 06510, United States; Section of Biomedical Informatics and Data Science, Yale School of Medicine, New Haven, CT 06510, United States; Center for Interdisciplinary Research on AIDS (CIRA), Yale School of Public Health, New Haven, CT 06510, United States; Division of Health Informatics, Department of Biostatistics, Yale School of Public Health, New Haven, CT 06510, United States; Division of Health Informatics, Department of Biostatistics, Yale School of Public Health, New Haven, CT 06510, United States; Department of Population Health Sciences, Health Analytics, Weill Cornell Medicine, New York, NY 10065, United States; Department of Biostatistics, Gillings School of Global Public Health, University of North Carolina at Chapel Hill, Chapel Hill, NC 27599, United States

**Keywords:** African Americans, women, anxiety, telemedicine, mHealth, mobile applications, digital health

## Abstract

**Objectives:**

This study aimed to understand Black American women’s attitudes toward seeking mental health services and using mobile technology to receive support for managing anxiety.

**Methods:**

A self-administered web-based questionnaire was launched in October 2019 and closed in January 2020. Women who identified as Black/African American were eligible to participate. The survey consisted of approximately 70 questions and covered topics such as, attitudes toward seeking professional psychological help, acceptability of using a mobile phone to receive mental health care, and screening for anxiety.

**Results:**

The findings of the study (*N* = 395) showed that younger Black women were more likely to have greater severity of anxiety than their older counterparts. Respondents were most comfortable with the use of a voice call or video call to communicate with a professional to receive support to manage anxiety in comparison to text messaging or mobile app. Younger age, higher income, and greater scores for psychological openness and help-seeking propensity increased odds of indicating agreement with using mobile technology to communicate with a professional. Black women in the Southern region of the United States had twice the odds of agreeing to the use of mobile apps than women in the Midwest and Northeast regions.

**Discussion:**

Black American women, in general, have favorable views toward the use of mobile technology to receive support to manage anxiety.

**Conclusion:**

Preferences and cultural appropriateness of resources should be assessed on an individual basis to increase likelihood of adoption and engagement with digital mental health interventions for management of anxiety.

## Introduction

Generalized anxiety disorder (GAD) is one of the most common mental health conditions among Black American women.^1^^,^^2^ Prior literature has shown that symptoms of GAD present differently in Black women^3^ and more chronically or severely when compared to their White counterparts.^4^ Archetypes deeply espoused by Black American women such as the Superwoman Schema^5^ and Strong Black Woman schema^6^ empower this population to demonstrate extraordinary resilience, independence, hard work, and strength, while commonly taking on the role of caregiver, despite adversity and systemically limited access to resources.^7–9^ However, such coping mechanisms have also shown to adversely influence attitudes toward seeking professional psychological services, and result in coping behaviors such as self-silencing or emotional suppression, which can lead to increased anxiety.^4^,^10–13^ Trauma, poor or toxic social and environmental conditions,^1^ health conditions such as premenstrual syndrome, pregnancy, and childbirth-related complications are common among Black women and have been found to increase the severity of anxiety symptoms.^14^ Such factors, in addition to gender and racial discrimination and wage inequities, establish a distinctive combination of contributing factors among this double-minority population, prompting the need for diagnostic and treatment tools tailored to providing culturally sensitive support to Black women with anxiety.^15^^,^^16^

Healthcare organizations significantly increased the use of telemental health services in response to the COVID-19 pandemic,^17^^,^^18^ solidifying telehealth services as an integral part of medical care.^17^ Research shows that telehealth services increase access to care, improve patient satisfaction, reduce patient costs, decrease no-show and cancelation rates, and improve patient-related outcomes such as treatment adherence.^17–22^ Black Americans reported the highest rate of telehealth services usage during the pandemic.^21^ However, more research is needed to understand which modalities are most effective for Black Americans, particularly given the digital inequities (eg, access to broadband internet) that impact the use of certain modalities such as video-enabled calls.^23^

Disparities in mental health service use frequently result from known barriers to seeking treatment (eg, stigma, lack of health insurance),^24–26^ which is concerning because this often leads to increased likelihood of developing or exacerbating depressive symptoms and poor management of anxiety symptoms. Consequently, the aforementioned factors can contribute to the development of physiological conditions such as high blood pressure, exacerbations in other cardiovascular risk factors, and impairments of the neurocircuitry associated with fear.^27^^,^^28^ Black women have high rates of smartphone ownership (80%)^29^ and, in general, are comfortable with participating in mobile health (mHealth) research and using digital tools for mental health support.^30–32^ Past interventions using modalities such as voice call,^33^^,^^34^ video call,^35^^,^^36^ text messaging,^37^ and mobile applications^38–40^ were effective in reducing anxiety symptoms. However, many of the published studies examining the use of mHealth interventions to support the management of anxiety were conducted with predominantly White participants. Therefore, the results may not be generalizable to all racial groups. Leveraging mobile technology may help to reduce barriers to receiving support by providing information on affordable options for mental health care, facilitating connections with preferred therapists, eliminating travel time through use of remote services, and reducing social stigma by providing a more discreet way to receive care.^41–43^

## Objective

This study aimed to understand Black American women’s attitudes toward seeking mental health services and using mobile technology to receive support for managing anxiety.

## Methods

The Institutional Review Board of the University of North Carolina at Chapel Hill (UNC IRB) provided the study a notification of exemption from further review. The self-administered web-based questionnaire was launched in October 2019 and closed in January 2020.^44^ Women (18 years or older) who identify as Black/African American (or multiracial, Black/African American and another race) and reside within the United States were eligible to participate. Participants were recruited through convenience sampling. Recruitment methods included receiving an invitation to take the survey via an anonymous link distributed through listservs whose membership is primarily Black American women (eg, National Council of Negro Women, Inc.), university student group listservs, and church membership listservs. Participants were also recruited via posts on social media (eg, Facebook, Twitter). Following a snowball sampling technique, respondents were allowed to share the link to the survey with their networks (eg, family, friends). Respondents that completed the survey were eligible to provide their contact information for entry into a drawing to receive one of 5 $100 eGift cards. Participation in the drawing was optional. The survey responses were collected and stored separately from the participants' identifiers. A separate questionnaire was used to collect contact information for the drawing and notification of an opportunity to participate in a focus group (participants opt-in to both). This questionnaire was linked to the end of the main survey.

Sample proportions from the preliminary study exploring the acceptability of telemedicine to support management of anxiety among Black American women were considered for use in nQuery statistical software to determine the necessary sample size to obtain a specified level of precision via the width of a 2-sided 95% CI of ±0.05.^30^^,^^45^ Based on a conservative scenario with an expected proportion of 0.50, 385 was determined to be the needed sample size to obtain this level of precision.

### Measures

The *Attitudes Toward Seeking Mental Health Services and Use of Mobile Technology Survey* has been approved for use by the UNC IRB (File S1). The survey consisted of approximately 70 questions and was administered using Qualtrics software. Survey domains included: (1) sociodemographic characteristics; (2) attitudes toward seeking professional psychological help (questions from an adapted version of the validated Inventory of Attitudes Toward Seeking Mental Health Services)^46^; (3) mobile phone use; (4) acceptability of using a mobile phone to receive mental health care; (5) screening for the presence and severity of anxiety using the GAD-7 scale^47^; (6) past mental health services utilization 2019 National Survey on Drug Use and Health (NSDUH)^48^; and (7) history of mental illness. Respondents were informed they could choose not to answer any question they do not wish to answer. They could also choose to stop taking the survey at any time.

Sociodemographic characteristics questions, such as the respondent’s race, ethnicity, age, gender, highest level of education attained, and annual household income were asked at the beginning of the survey. The race, age, and gender questions were used as screener questions to determine eligibility to continue the survey. If the respondent did not self-identify as Black/African American (or multiracial, Black/African American, and another race) and 18 years or older, they were routed directly to the end of the survey. Also, if the respondent self-identified as male, they were routed directly to the end of the survey. A detailed description of the measures is provided in File S2. To prevent false responses from bots, the aforementioned screener questions were used in the skip logic for the web-based questionnaire (routed to the end of survey) and the options to prevent multiple submissions and prevent indexing were active. During the time of data collection, the first author reviewed the data at least twice per week for suspicious responses.

### Statistical analysis

#### Quantitative data analysis

Descriptive statistics were calculated as means, standard deviations, and ranges for continuous variables, and as frequencies and percentages for categorical variables for sample characteristics and responses to questions about modality use (eg, video call) to receive mental health services. The age variable was grouped (18-24, 25-34, 35-49, 50-64, over 65 years) according to results from a prior study on the mental health of Black women in the United States.^1^ Demographic variables also included race (Black or African American, multiracial defined as identifying as Black or African American and at least 1 other race), ethnicity (Hispanic, non-Hispanic), education (high school diploma or GED, some college less than 4-year degree, Bachelor’s degree or higher), income (less than $10 000, $10 000-24 999, $25 000-49 999, $50 000-100 000, more than $100 000), health insurance (yes, no), and region of the United States in which the respondent currently lives (Midwest, Northeast, West, South). Additionally, age was dichotomized into 2 groups (less than 50 years and 50 or older) and education was categorized into 2 levels (less than Bachelor’s degree and Bachelor’s degree or higher) as reported in the preliminary study.^30^^,^^45^

Response options were dichotomized as agreed (*agree/somewhat agree*) versus those who did not indicate agreement (*disagree/somewhat disagree/undecided*). Fisher’s exact test was used to determine whether a statistically significant association exists between response to questions about comfortability with using each modality to communicate with a professional to receive help for managing anxiety (agreed vs those who did not indicate agreement), with age group and education level, respectively. Independent groups *t-*tests were performed separately to assess group differences in mean scores for GAD-7, psychological openness, help-seeking propensity, indifference to anxiety stigma, and IASMHS scores for anxiety, between the groups of participants who agreed with use of various modalities to communicate with a professional to receive help to manage anxiety versus those who did not indicate agreement. Missing responses were excluded from analysis.

Multivariable logistic regression models were performed to estimate odds ratios (OR) and corresponding 95% CI to assess whether an association exists between the responses to questions about comfortability with using each modality to communicate with a professional to receive help for managing anxiety (agreed vs those who did not indicate agreement), with education level, household income, health insurance, GAD-7 score, psychological openness, help-seeking propensity, indifference to anxiety stigma, past mental health service use, unmet mental health need, and region, respectively. Age and history of anxiety were managed as control variables. Statistical significance was determined at the 2-sided .05 significance level for all tests; no adjustments were made for multiple comparisons. Statistical analyses were conducted using both SPSS and SAS statistical software.

## Results

The results of this article focus on Black American women’s attitudes toward use of mobile technology to support the management of anxiety. The sociodemographic characteristics of the survey respondents are summarized in Table 1. Three hundred ninety-five completed the survey out of the 491 respondents that started the survey (80.4% completion rate). Respondents ranged in age from 18 to 107 years (mean age of 44.8 ± SD 18.4 years), and all identified as either Black/African American or multiracial (ie, Black/African American and another race) and female. Most respondents (96.2%) identified as non-Hispanic. Furthermore, 79.0% obtained a bachelor’s degree or higher. Annual household income was reported as less than $50 000 for 40.3%, $50 000-$100 000 for 34.9%, and more than $100 000 for 23.8% of respondents. The majority (93.9%) indicated that they had health insurance.

**Table 1.** Sociodemographic characteristics of the survey respondents (*N* = 395).

Age (years), mean (SD)	44.8 (18.4)
Age group (years), *n* (%)	
18-24	59 (14.9)
25-34	98 (24.8)
35-44	46 (11.6)
45-54	58 (14.7)
55-64	55 (13.9)
65 and older	79 (20.0)
Age group (years), *n* (%)	
Less than 50	232 (58.7)
50 or older	163 (41.3)
Race, *n* (%)	
Black or African American	377 (95.4)
Multiracial^a^	18 (4.6)
Ethnicity, *n* (%)	
Hispanic	15 (3.8)
Non-Hispanic	380 (96.2)
Education, *n* (%)	
High school diploma or GED	13 (3.3)
Some college, less than 4-year degree	70 (17.7)
Bachelor’s degree or higher	312 (79.0)
Education, *n* (%)	
Less than bachelor’s degree	83 (21.0)
Bachelor’s degree or higher	312 (79.0)
Income,^b^ *n* (%)	
Less than $10 000	30 (7.6)
$10 000-24 999	37 (9.4)
$25 000-49 999	92 (23.3)
$50 000-100 000	138 (34.9)
More than $100 000	94 (23.8)
Health Insurance,^c^ *n* (%)	
Yes	371 (93.9)
No	23 (5.8)
Region, *n* (%)	
Midwest	60 (15.19)
Northeast	69 (17.47)
West	34 (8.61)
South	229 (57.97)

a Multiracial defined as identifying as Black or African American and at least one other race.

b Total *N* less than 395 and percentages may not sum to 100% because of item missingness (*n* = 4) and rounding.

c Total *N* less than 395 and percentages may not sum to 100% because of item missingness (*n* = 1) and rounding.

Most respondents (98.2%) reported use of text messaging, and 72.7% indicated texting 4 or more times per day. The majority (96.4%) indicated that they use mobile apps, and 73.4% indicated using a mobile app 4 or more times per day. Furthermore, many (80.5%) reported use of video call, and 38.4% indicated using video call at least 1 time per week.

### Anxiety severity

**Figure 1.**
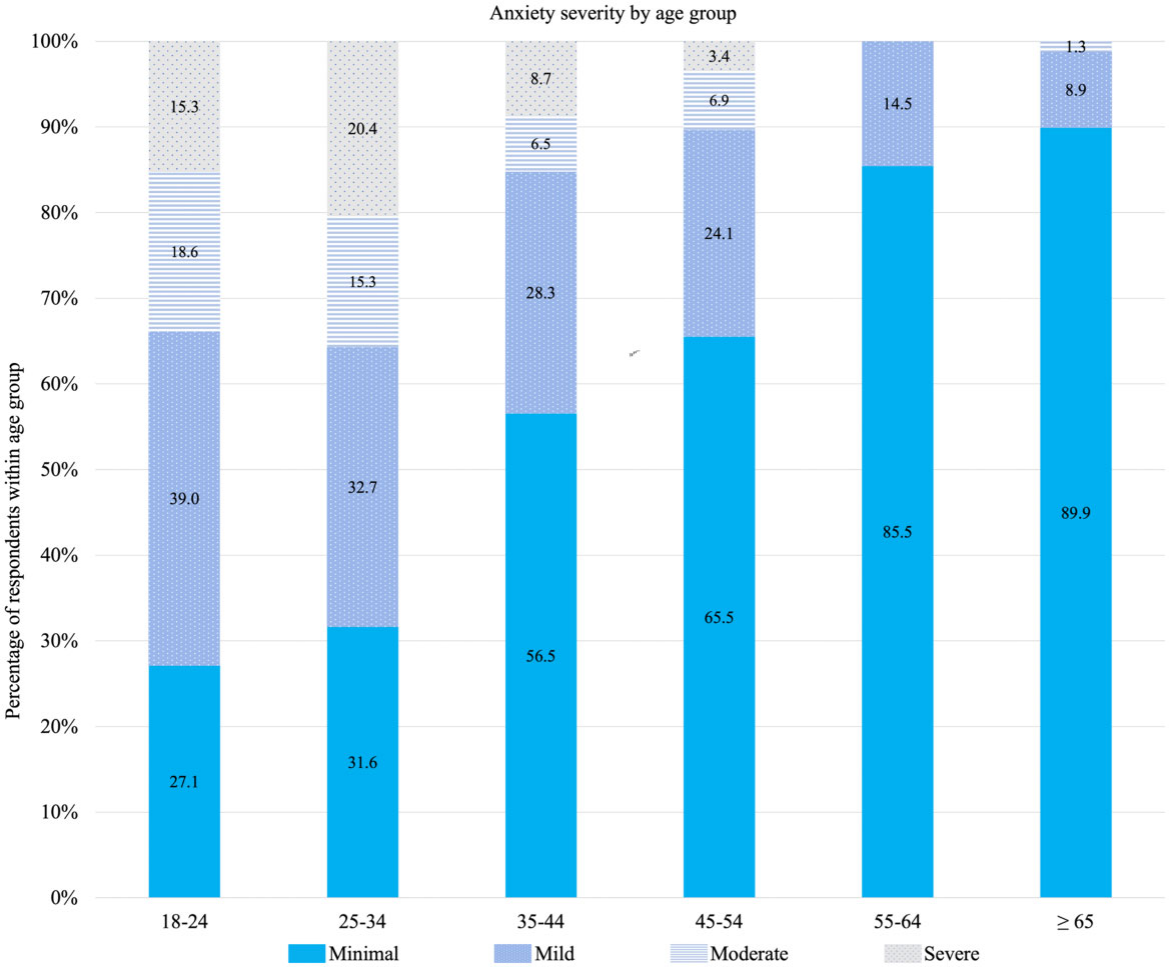
Sample percentages for anxiety severity by age group.

Approximately, 29% of respondents reported being diagnosed with anxiety in the past. Questions from the GAD-7 were asked to screen for current presence and severity of anxiety.^47^ The mean score for anxiety severity (GAD-7) was 4.86 (SD 5.29) on a scale of 0 (minimal) to 21 (severe). The sample percentages for anxiety severity by age group are displayed in Figure 1. The 25- to 34-year-old age group had the highest percentage of anxious individuals, reporting 15.3% of respondents with moderate and 20.4% of respondents with severe level of anxiety. Respondents in the 18- to 24-year-old age group had the next highest percentage of anxious individuals, reporting 18.6% of respondents with moderate and 15.3% of respondents with severe level of anxiety. The 35- to 44-year-old age group reported 6.5% of respondents with moderate and 8.7% of respondents with severe level of anxiety. The 45- to 54-year-old age group reported 6.9% of respondents with moderate and 3.4% of respondents with severe level of anxiety. The 55- to 64-year-old age group reported no respondents with moderate or severe level of anxiety. Lastly, the 65 and older age group reported 1.3% of respondents with moderate and no respondents with severe level of anxiety.

### The acceptability of mobile technology to support the management of anxiety

**Figure 2.**
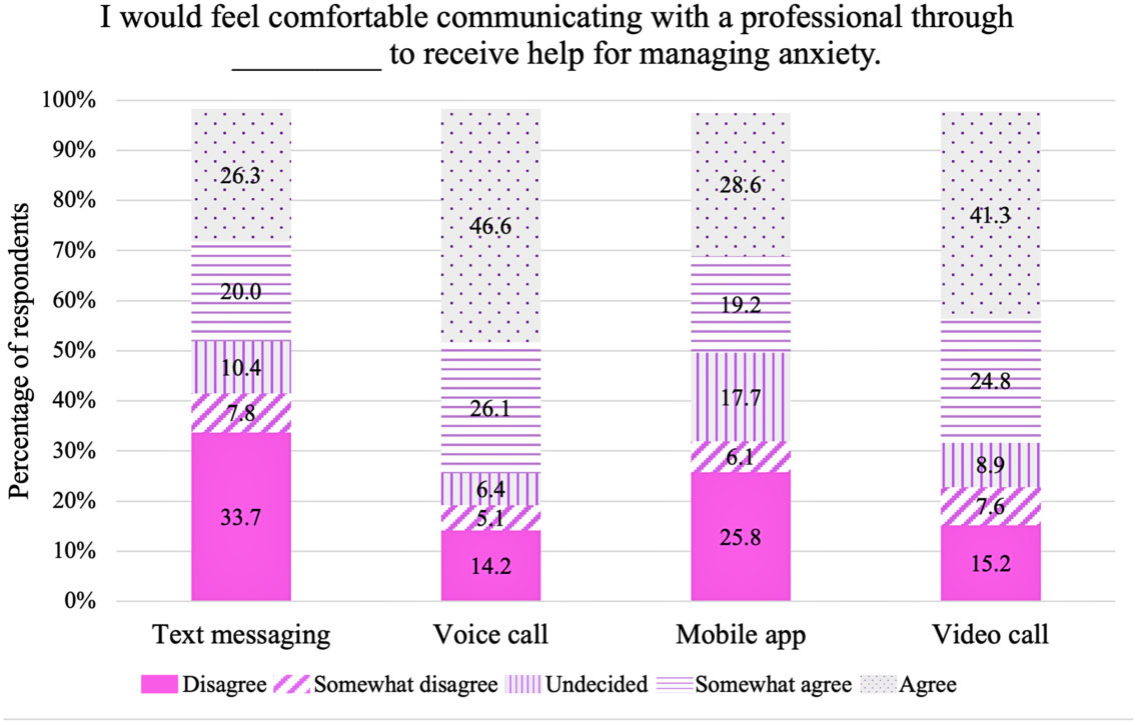
Sample percentages, by modality, for response to the statement, “I would feel comfortable communicating with a professional through [modality] to receive help for managing anxiety.”

**Figure 3.**
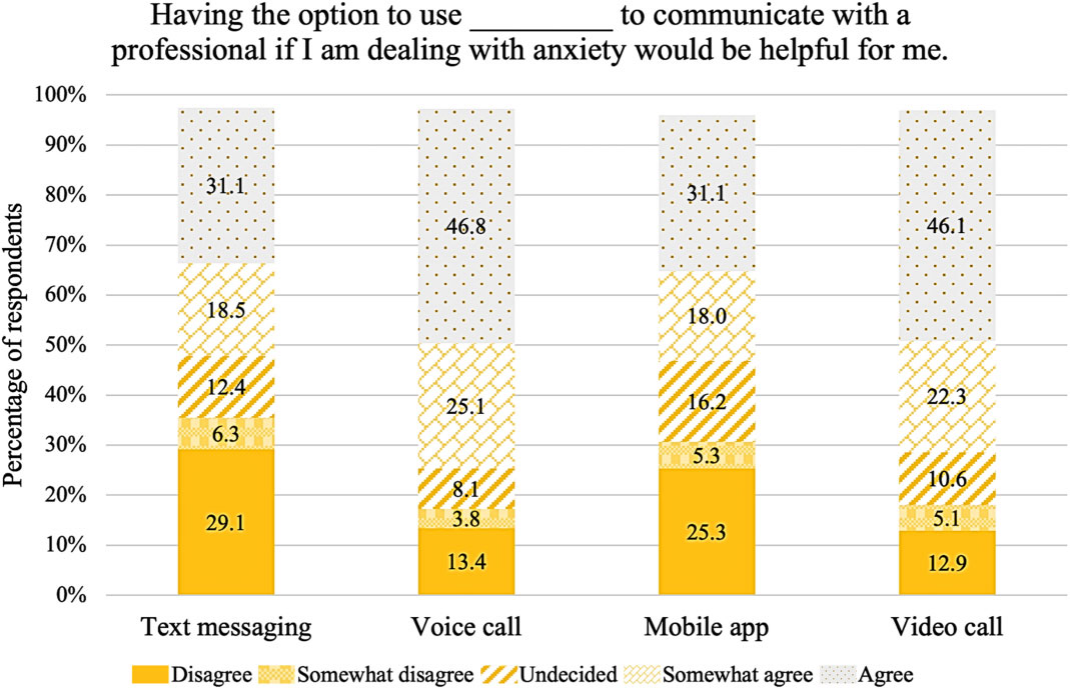
Sample percentages, by modality, for response to the statement, “Having the option to use [modality] to communicate with a professional if I am dealing with anxiety would be helpful for me.”

The results of the survey revealed that respondents were most comfortable with the use of a voice call, followed by video call, mobile app, and lastly, text messaging to communicate with a professional to receive support to manage anxiety (Figure 2). Approximately 73% of respondents indicated agreement (46.6% agree and 26.1% somewhat agree), 6.3% were undecided, and 19.3% showed disagreement (14.2% disagree and 5.1% somewhat disagree) with the use of a voice call to receive support for managing anxiety. Furthermore, 66.1% of respondents indicated agreement (41.3% agree and 24.8% somewhat agree), 8.9% were undecided, and 22.8% showed disagreement (15.2% disagree and 7.6% somewhat disagree) with the use of a video call to receive support for managing anxiety. Approximately 48% of respondents indicated agreement (28.6% agree and 19.2% somewhat agree), 17.7% were undecided, and 31.9% showed disagreement (25.8% disagree and 6.1% somewhat disagree) with the use of a mobile app to receive support for managing anxiety. Similarly, 46.3% of respondents indicated agreement (26.3% agree and 20.0% somewhat agree), 10.4% were undecided, and 41.5% showed disagreement (33.7% disagree and 7.8% somewhat disagree) with the use of text messaging to receive support for managing anxiety. Approximately 72% of respondents agreed (46.8% agree and 25.1% somewhat agree) that having the option to use voice call to communicate with a professional if they are dealing with anxiety would be helpful. Furthermore, 68.4% of respondents agreed (46.1% agree and 22.3% somewhat agree) that a video call option would be helpful, 49.1% agreed (31.1% agree and 18.0% somewhat agree) that a mobile app option would be helpful, and 49.6% agreed (31.1% agree and 18.5% somewhat agree) that a text messaging option would be helpful (Figure 3).

Statistically significant associations were found between age (less than 50 years old vs 50+) and agreement (agree/somewhat agree) with the use of text messaging (55.2% agreed vs 33.7% agreed, respectively, *P* < .001), mobile app (58.2% agreed vs 33.1% agreed, respectively, *P* < .001), and video call (74.1% agreed vs 54.6% agreed, respectively, *P* = .001) to communicate with a professional to receive support for managing anxiety (Table 2). No statistically significant association was found between age (less than 50 years old vs 50+) and agreement (agree/somewhat agree) with the use of voice call to communicate with a professional to receive support for managing anxiety (76.3% agreed vs 67.5% agreed, respectively, *P* = .238). Furthermore, no statistically significant associations were found between education (less than Bachelor’s vs Bachelor’s degree or higher) and agreement (agree/somewhat agree) with the use of text messaging (54.2% agreed vs 44.2% agreed, respectively, *P* = .104), voice call (71.1% agreed vs 73.1% agreed, respectively, *P* = .778), mobile app (56.6% agreed vs 45.5% agreed, respectively, *P* = .060), and video call (61.4% agreed vs 67.3% agreed, respectively, *P* = .350) to communicate with a professional to receive help for managing anxiety (Table 3).

**Table 2.** Agreement with use of modality to communicate with a professional to receive help for managing anxiety by age group.

Modality^a^	Less than 50 years old (*N* = 232)		50 years and older (*N* = 163)		Fisher’s exact *P* value
	Agree, *N* (%)	No agree, *N* (%)	Agree, *N* (%)	No agree, *N* (%)	
Text messaging	128 (55.2)	104 (44.8)	55 (33.7)	101 (62.0)	**<.001**
Voice call	177 (76.3)	55 (23.7)	110 (67.5)	46 (28.2)	.238
Mobile app	135 (58.2)	97 (41.8)	54 (33.1)	99 (60.7)	**<.001**
Video call	172 (74.1)	60 (25.9)	89 (54.6)	65 (39.9)	**.001**

a Total *N* less than 395 and percentages may not sum to 100% due to item missingness. Note: *Undecided* responses were combined with disagree and somewhat disagree responses to form a category of “no agree” for those who did not indicate agreement. Bolded *P* value denotes statistical significance.

**Table 3.** Agreement with use of modality to communicate with a professional to receive help for managing anxiety by education.

Modality^a^	Less than bachelor’s degree (*N* = 83)		Bachelor’s degree or higher (*N* = 312)		Fisher’s exact *P* value
	Agree, *N* (%)	No agree, *N* (%)	Agree, *N* (%)	No agree, *N* (%)	
Text messaging	45 (54.2)	36 (43.4)	138 (44.2)	169 (54.2)	.104
Voice call	59 (71.1)	22 (26.5)	228 (73.1)	79 (25.3)	.778
Mobile app	47 (56.6)	33 (39.8)	142 (45.5)	163 (52.2)	.060
Video call	51 (61.4)	30 (36.1)	210 (67.3)	95 (30.4)	.350

a Total *N* less than 395 and percentages may not sum to 100% due to item missingness. Note: *Undecided* responses were combined with disagree and somewhat disagree responses to form a category of “no agree” for those who did not indicate agreement.

### Attitudes toward seeking help by agreement with modality

The results of the study showed statistically significant differences between group mean scores for GAD-7 (5.98 ± SD 5.72 vs 3.91 ± SD 4.74, respectively, *P* < .001) and indifference to anxiety stigma (24.46 ± 6.44 vs 25.92 ± 5.50, respectively, *P* = .018) between the participants who agreed with the use of text messaging to communicate with a professional to receive help to manage anxiety and those who did not indicate agreement (File S3). No statistically significant differences were found between group mean scores for psychological openness (23.27 ± 5.71 vs 23.78 ± 5.40, respectively, *P* = .367), help-seeking propensity (25.97 ± 5.09 vs 25.79 ± 5.96, respectively, *P* = .758), and IASMHS total score (73.68 ± 13.96 vs 75.38 ± 13.51, respectively, *P* = .228) between the participants who agreed with the use of text messaging to communicate with a professional to receive help to manage anxiety and those who did not indicate agreement. Likewise, no statistically significant differences were found between group mean scores for GAD-7 (4.85 ± 5.28 vs 5.02 ± 5.46, respectively, *P* = .787), psychological openness (23.54 ± 5.54 vs 23.46 ± 5.60, respectively, *P* = .906), help-seeking propensity (26.19 ± 5.37 vs 24.95 ± 6.01, respectively, *P* = .070), indifference to anxiety stigma (25.22 ± 6.04 vs 25.18 ± 5.85, respectively, *P* = .956), and IASMHS total score (74.89 ± 13.62 vs 73.44 ± 14.01, respectively, *P* = .374) between the participants who agreed with the use of voice call to communicate with a professional to receive help to manage anxiety and those who did not indicate agreement (File S3).

Moreover, statistically significant differences were found in group mean scores for GAD-7 (5.78 ± 5.58 vs 4.13 ± 4.95, respectively, *P* = .002) between the participants who agreed with the use of mobile app to communicate with a professional to receive help to manage anxiety and those who did not indicate agreement (File S3). However, no statistically significant differences were found between group mean scores for psychological openness (23.32 ± 5.67 vs 23.62 ± 5.43, respectively, *P* = .600), help-seeking propensity (25.72 ± 5.78 vs 25.96 ± 5.36, respectively, *P* = .681), indifference to anxiety stigma (24.75 ± 6.37 vs 25.64 ± 5.61, respectively, *P* = .145), and IASMHS total score (73.79 ± 14.38 vs 75.07 ± 13.01, respectively, *P* = .361) between the participants who agreed with the use of mobile app to communicate with a professional to receive help to manage anxiety and those who did not indicate agreement. Statistically significant differences were found between group mean scores for psychological openness (23.95 ± 5.44 vs 22.51 ± 5.66, respectively, *P* = .019), help-seeking propensity (26.23 ± 5.23 vs 24.98 ± 6.11, respectively, *P* = .049), and IASMHS total score (75.46 ± 12.95 vs 72.24 ± 14.92, respectively, *P* = .041) between the participants who agreed with the use of video call to communicate with a professional to receive help to manage anxiety and those who did not indicate agreement (File S3). No statistically significant differences were found between group mean scores for GAD-7 (5.13 ± 5.45 vs 4.55 ± 5.03, respectively, *P* = .305) and indifference to anxiety stigma (25.34 ± 5.96 vs 24.84 ± 6.06, respectively, *P* = .447) between the participants who agreed with the use of video call to communicate with a professional to receive help to manage anxiety and those who did not indicate agreement.

The results of the multivariable logistic regression analysis showed a statistically significant odds ratio for respondents who agreed to using text messaging to communicate with a professional to receive help for managing anxiety with household income ($25 000-49 999 vs Less than $25 000) (adjusted OR 2.96, 95% CI 1.46-5.99). No statistically significant odds ratios were shown between respondents who agreed to using text messaging to communicate with a professional to receive help for managing anxiety and education level, household income, health insurance, GAD-7, psychological openness, help-seeking propensity, indifference to anxiety stigma, past mental health service use, unmet mental health need, or region (File S4). No statistically significant odds ratios were shown between respondents who agreed to using voice call to communicate with a professional to receive help for managing anxiety and education level, household income, health insurance, GAD-7, psychological openness, help-seeking propensity, indifference to anxiety stigma, past mental health service use, unmet mental health need, or region (File S4).

Statistically significant odds ratios were found between respondents’ who agreed to using mobile apps to communicate with a professional to receive help for managing anxiety and region (midwest vs south; adjusted OR 0.50, 95% CI 0.27-0.91) and region (northeast vs south; adjusted OR 0.55, 95% CI 0.31-0.99). No statistically significant odds ratios were found between the respondents’ who agreed to using video call to communicate with a professional to receive help for managing anxiety and education level, household income, health insurance, GAD-7, psychological openness, help-seeking propensity, indifference to anxiety stigma, past mental health service use, or unmet mental health need (File S4).

Statistically significant odds ratios were found between respondents’ who agreed to using video call to communicate with a professional to receive help for managing anxiety and income (more than $100 000 vs less than $25 000; adjusted OR 3.34, 95% OR 1.49-7.49), GAD-7 (score 10-21 vs score 0-9; adjusted OR 0.51, 95% OR 0.27-0.97), psychological openness (score 17-32 vs score 0-16) (adjusted OR 2.14, 95% CI 1.12-4.11), and help-seeking propensity (score 17-32 vs score 0-16; adjusted OR 3.50, 95% CI 1.50-8.16). No statistically significant associations were found among respondents who agreed to using mobile apps to communicate with a professional to receive help for managing anxiety and education level, health insurance, indifference to anxiety stigma, past mental health service use, unmet mental health need, or region (File S4). The primary concerns were privacy and confidentiality, communication issues (eg, misinterpreting text), and the impersonal feeling of communicating by mobile phone (eg, SMS text messaging) with regard to using mobile technology to receive support.^44^

## Discussion

Findings from this study revealed higher prevalence of anxiety among survey respondents (28.9%) compared to that reported among non-Hispanic Black women in a large national survey (15.7%).^2^ Moreover, the results showed that younger Black American women were more likely to have higher levels of anxiety than their older counterparts. The 25- to 34-year-old age group, followed by the 18- to 24-year-old age group, had the highest percentage of individuals with moderate or severe anxiety compared to the older age groups. The results showed a trend of anxiety severity gradually decreasing among the older age groups. This finding is consistent with prior literature that reported that younger Black women (less than 50 years old) had higher prevalence of lifetime anxiety disorders (eg, general anxiety disorder) than older Black women.^1^ We hypothesize that older Black women may present with less symptoms of anxiety due to learning how to navigate stressors over time.^49^

Respondents were most comfortable with the use of a voice call or video call to communicate with a professional to receive help to manage anxiety. Furthermore, most respondents agreed that the option to use voice call or video call to communicate with a professional would be helpful for them. Younger Black women (less than 50 years old) were significantly more likely to endorse use of text messaging, mobile apps, and video call. Respondents who agreed to use voice call, video call, or text messaging were typically more anxious than those who did not agree. Also, respondents who agreed with the use of text messaging, as a group, had greater indifference to anxiety stigma than the group who did not indicate agreement. Results were consistent with findings from the preliminary study.^30^^,^^45^

No significant findings emerged in regard to education. It is worth noting that the number of participants with a high school diploma or GED was collapsed into the “Less than bachelor’s degree” category. Although the numbers for Bachelor’s degree or higher are significantly greater, participants with some college may have skewed the results masking significant differences between the groups. Future studies may benefit from evaluating differences between high school or equivalent experiences, college experience, and graduate education in mental health services. Previous studies have shown that higher education is typically associated with greater help-seeking behavior.^50^^,^^51^

Key findings from the multivariable logistic regression analysis revealed that respondents with a household income of $25 000-$49 999 have 3 times the odds of agreement with using text messaging to communicate with a professional to receive support to manage anxiety compared to respondents who reported an income less than $25 000. Additionally, the results revealed increased odds of agreement to using mobile apps to communicate with a professional to receive support to manage anxiety among respondents in the Southern region of the United States compared to respondents in the Midwest and Northeast regions, by approximately twice the odds. Lower scores for anxiety severity (vs higher scores) revealed increased odds of agreement with using video calls to communicate with a professional to receive support to manage anxiety by twice the odds. Conversely, higher scores for psychological openness and help-seeking propensity increased odds of indicating agreement with using video calls to communicate with a professional to receive support to manage anxiety, by approximately 2-3 times the odds.

### Limitations

The main limitations were the mode used to administer the survey and the recruitment methods. Due to the sensitive nature of the questions and for convenience, the computer-assisted web interviewing data collection technique was used to administer the survey. Although this method may increase privacy and reduce respondent burden in completing the survey, individuals who do not have email access or a social media account may not be able to complete the survey by accessing the link. Regarding recruitment, participants were recruited through convenience sampling and encouraged to share the survey email or social media posts with their networks. No personally identifiable information was collected in the survey and respondents accessed the survey through an anonymous link; however, social desirability bias could have resulted if the respondent personally knew the PI (TM). In addition, the sample consisted of mostly highly educated women (79%), with health insurance, at rates higher than the general population of Black women (28%).^52^ Therefore, our ability to generalize the findings to all Black American women with anxiety is limited. With regard to our use of the GAD-7 to assess the presence and severity of anxiety, the instrument may be limited in its ability to identify the complete range of relevant anxiety symptoms among Black women. The GAD-7 was used in the study for comparison with results from the preliminary study.^53^ However, prior research has shown that there is a need for more culturally sensitive GAD screening tools.^53^

## Conclusion and future directions

This study was conducted before the COVID-19 pandemic. In general, Black women endorsed the use of mobile technology, with voice calls and video calls being the most preferred modalities to receive support for managing anxiety. The Black community was disproportionately affected by COVID-19 infections and death, job loss, under/unemployment,^54^ and psychological distress exacerbated by increased racial violence^55^; factors which had deleterious effects on the mental health of Black women. There is a great need to provide more affordable, accessible, and culturally relevant mental health services and resources to this population. This article contributes to research on cultural sensitivity in the assessment of digital mental interventions for the management of anxiety. Preferences and cultural appropriateness of resources should be assessed on an individual basis to increase the likelihood of adoption and engagement with digital mental health interventions for the management of anxiety. Future work will include the development and implementation of mHealth interventions and digital mental health tools for Black women, such as a mobile app to support management of anxiety among young Black American women.^43^^,^^56^

## Supplementary Material

ooad088_Supplementary_Data

## Data Availability

The raw data supporting the conclusions of this article are available from the Dryad Digital Repository: https://doi.org/10.5061/dryad.sn02v6x9t (2023).
