# Supplementary material for: Black American women’s attitudes toward seeking mental health services and use of mobile technology to support the management of anxiety
Source: JAMIA Open. 2023 Oct 17;6(4):ooad088. doi: 10.1093/jamiaopen/ooad088 (PMC10582519; doi:10.1093/jamiaopen/ooad088)
Supplement: ooad088_Supplementary_Data [file ooad088_supplementary_data.zip › Supplementary_File_2_Description of Measures.pdf]

Supplementary File 2: Description of Measures

Anxiety Screening

If respondents passed the screening questions, they were permitted to complete the rest of the survey. The next set of questions from the Generalized Anxiety Disorder (GAD-7) scale [31] were included in the survey to screen for the presence and severity of anxiety. The GAD-7 scale is a seven-item measure evaluating the presence and severity of generalized anxiety disorder. The scale ranges from a score of 0 to 21. A score of ≥ 10 on the GAD-7 represents a reasonable cut-point for identifying cases of GAD Scores of 0 to 4 indicate *minimal*, 5 to 9 *mild*, 10 to 14 *moderate*, and 15 to 21 *severe* level of anxiety.

Attitudes Toward Seeking Professional Psychological Help

Respondents' attitudes toward seeking professional psychological help were measured using questions from an adapted version of the validated *Inventory of Attitudes Toward Seeking Mental Health Services* (IASMHS) The IASMHS consists of 24 questions that contribute to a total IASMHS score and the following factors: psychological openness (i.e., the extent to which individuals are open to acknowledging psychological problems and to the possibility of seeking professional help for them), help-seeking propensity (i.e., the extent to which individuals believe they are willing and able to seek professional psychological help), and indifference to stigma (i.e., the extent to which individuals are concerned about what various important others might think should they find out that the individual were seeking professional help for psychological problems). Response options to the survey items were on a 5-point Likert-type scale ranging from 0 (disagree) to 4 (agree). Before data analysis, all negatively worded items were reverse coded.

In the survey, the term *professional* referred to individuals who are trained mental health specialists (e.g., psychologists, psychiatrists, social workers, and family physicians). To collect

data specifically about attitudes toward seeking professional help for managing anxiety and depression, six questions in the inventory were revised. In these six questions, the words *psychological problems* or *mental disorder* were substituted with *anxiety*. For example, item #16 in the IASMHS reads, “I would be uncomfortable seeking professional help for psychological problems because people in my social or business circles might find out about it.” The revised survey question states, “I would be uncomfortable seeking professional help for *anxiety* because people in my social or business circles might find out about it.” This increased the total number of questions in the inventory and permitted calculation of a total IASMHS score related to anxiety, and subscores for psychological openness, help-seeking propensity, and indifference to stigma for anxiety. Scores on the IASMHS range from 0 to 96, with subscale scores ranging from 0 to 32. Higher scores indicate more positive attitudes toward seeking professional psychological help.

Mental Health Service Utilization

Data on past mental health services use was collected through use of questions from the *2019 National Survey on Drug Use and Health (NSDUH)* [32]. Respondents were given instructions that the questions were about treatment and counseling for problems with emotions, nerves or mental health. They were also asked not to include treatment for alcohol or drug use.

Mobile Phone Use

Mobile phone use was ascertained with the following items: (1) current mobile phone ownership (yes/no); (2) frequency of sending text messages (never, less than 1 time per week, 1-6 times per week, 1-3 times per day, 4 or more times per day); (3) frequency of accessing apps on phone (never, less than 1 time per week, 1-6 times per week, 1-3 times per day, 4 or more times per day); (4) ability to complete video calls on mobile phone (yes/no); and (5) frequency of using mobile phone to complete video calls (never, less than 1 time per week, 1-6 times per week, 1-3 times per

day, 4 or more times per day).

The Acceptability of Mobile Phone Use for Mental Health Care

The acceptability of using a mobile phone to receive mental health care to manage anxiety was measured by responses to questions regarding comfortability with communicating with a professional through [text messaging/voice call/mobile app/video call] to receive help for managing anxiety. Respondents were also asked whether, “Having the option to use [text messaging/voice call/mobile app/video call] to communicate with a professional if I am dealing with anxiety would be helpful for me.” Response options to the survey items were on a 5-point Likert-type scale ranging from 1 (disagree) to 5 (agree).

Mental Health History

The final section of the survey asked questions about past diagnosis of anxiety. Respondents were also asked if they currently have health insurance. If so, they were presented with the question, “Does your health insurance pay for any type of mental health treatment or counseling services?”
