# Supplementary material for: Black American women’s attitudes toward seeking mental health services and use of mobile technology to support the management of anxiety
Source: JAMIA Open. 2023 Oct 17;6(4):ooad088. doi: 10.1093/jamiaopen/ooad088 (PMC10582519; doi:10.1093/jamiaopen/ooad088)
Supplement: ooad088_Supplementary_Data [file ooad088_supplementary_data.zip › Supplementary_File_3_Attitudes Toward Seeking Support by Agreement with Modality.pdf]

Supplementary File 3: Attitudes Toward Seeking Support by Agreement with Modality

Generalized Anxiety Disorder (GAD-7) and Inventory of Attitudes Toward Seeking Mental Health Services factor scores by agreement with using **text messaging** to communicate with a professional to receive help for managing **anxiety**

	Agree		No Agree				
Measure*	N	Mean score (SD)	N	Mean score (SD)	t	df	p-value
GAD-7	183	5.98 (5.72)	205	3.91 (4.74)	3.86	354.69	**< .001**
Psychological openess	183	23.27 (5.71)	204	23.78 (5.40)	-.90	374.94	.367
Help-seeking propensity	183	25.97 (5.09)	204	25.79 (5.96)	.31	384.12	.758
Indifference to anxiety stigma	182	24.46 (6.44)	203	25.92 (5.50)	-2.37	357.83	**.018**
IASMHS total	182	73.68 (13.96)	202	75.38 (13.51)	-1.21	374.93	.228

*Total N less than 395 and percentages may not sum to 100% due to item missingness. Note: *Undecided* responses were combined with Disagree and Somewhat disagree responses to form a category of "No Agree" for those who did not indicate agreement.

Generalized Anxiety Disorder (GAD-7) and Inventory of Attitudes Toward Seeking Mental Health Services factor scores by agreement with using **voice call** to communicate with a professional to receive help for managing **anxiety**

	Agree		No Agree				
Measure*	N	Mean score (SD)	N	Mean score (SD)	t	df	p-value
GAD-7	287	4.85 (5.28)	101	5.02 (5.46)	-.27	170.12	.787
Psychological openess	287	23.54 (5.54)	100	23.46 (5.60)	.12	171.20	.906
Help-seeking propensity	287	26.19 (5.37)	100	24.95 (6.01)	1.82	157.49	.070
Indifference to anxiety stigma	285	25.22 (6.04)	100	25.18 (5.85)	.05	178.39	.956
IASMHS total	285	74.89 (13.62)	99	73.44 (14.01)	.89	166.63	.374

*Total N less than 395 and percentages may not sum to 100% due to item missingness. Note: *Undecided* responses were combined with Disagree and Somewhat disagree responses to form a category of "No Agree" for those who did not indicate agreement.

Generalized Anxiety Disorder (GAD-7) and Inventory of Attitudes Toward Seeking Mental Health Services factor scores by agreement with using **mobile app** to communicate with a professional to receive help for managing **anxiety**

	Agree		No Agree				
Measure*	N	Mean score (SD)	N	Mean score (SD)	t	df	p-value
GAD-7	189	5.78 (5.58)	195	4.13 (4.95)	3.06	373.47	**.002**
Psychological openess	189	23.32 (5.67)	195	23.62 (5.43)	-.53	379.90	.600
Help-seeking propensity	189	25.72 (5.78)	195	25.96 (5.36)	-.41	377.77	.681
Indifference to anxiety stigma	189	24.75 (6.37)	193	25.64 (5.61)	-1.46	371.83	.145
IASMHS total	189	73.79 (14.38)	192	75.07 (13.01)	-.91	374.06	.361

*Total N less than 395 and percentages may not sum to 100% due to item missingness. Note: *Undecided* responses were combined with Disagree and Somewhat disagree responses to form a category of “No Agree” for those who did not indicate agreement.

Generalized Anxiety Disorder (GAD-7) and Inventory of Attitudes Toward Seeking Mental Health Services factor scores by agreement with using **video call** to communicate with a professional to receive help for managing **anxiety**

	Agree		No Agree				
Measure*	N	Mean score (SD)	N	Mean score (SD)	t	df	p-value
GAD-7	260	5.13 (5.45)	125	4.55 (5.03)	1.03	263.08	.305
Psychological openess	260	23.95 (5.44)	125	22.51 (5.66)	2.37	236.20	**.019**
Help-seeking propensity	260	26.23 (5.23)	125	24.98 (6.11)	1.98	213.87	**.049**
Indifference to anxiety stigma	259	25.34 (5.96)	124	24.84 (6.06)	.76	238.74	.447
IASMHS total	258	75.46 (12.95)	124	72.24 (14.92)	2.06	214.81	**.041**

*Total N less than 395 and percentages may not sum to 100% due to item missingness. Note: *Undecided* responses were combined with Disagree and Somewhat disagree responses to form a category of “No Agree” for those who did not indicate agreement.
