# Supplementary material for: Black American women’s attitudes toward seeking mental health services and use of mobile technology to support the management of anxiety
Source: JAMIA Open. 2023 Oct 17;6(4):ooad088. doi: 10.1093/jamiaopen/ooad088 (PMC10582519; doi:10.1093/jamiaopen/ooad088)
Supplement: ooad088_Supplementary_Data [file ooad088_supplementary_data.zip › Supplementary_File_4_ MLR_ Attitudes Toward Using Mobile Technology.pdf]

Supplementary File 4: Multivariable Logistic Regression Models for Attitudes Toward Using Mobile Technology to Communicate with a Professional to Receive Support for Managing Anxiety By Modality

Multivariable logistic regression models for attitudes toward using **text messaging** to communicate with a professional to receive support for managing **anxiety.**

	Agree[a]	Unadjusted OR (95% CI)	Age-adjusted[b] OR (95% CI)	Multivariably adjusted[c] OR (95% CI)	p-value for pairwise comparison vs. reference
Age					
18-24	34/59 (57.6%)	Ref[f]	N/A	N/A	N/A
25-34	52/98 (53.1%)	0.831 (0.433-1.594)	N/A	N/A	N/A
35-44	30/46 (65.2%)	1.379 (0.622-3.058)	N/A	N/A	N/A
45-54	24/58 (41.4%)	0.519 (0.249-1.082)	N/A	N/A	N/A
55-64	14/55 (25.5%)	**0.264 (0.119-0.587)**	N/A	N/A	N/A
65+	29/79 (36.7%)	**0.474 (0.236-0.950)**	N/A	N/A	N/A
Age					
Less than 50	55/163 (33.7%)	Ref	N/A	N/A	N/A
50+	128/232 (55.2%)	**0.442 (0.291-0.672)**	N/A	N/A	N/A
Education					
Less than bachelor's degree	138/312 (44.2%)	Ref	Ref	Ref	Ref
Bachelor's degree or higher	45/83 (54.2%)	0.653 (0.399-1.069)	0.708 (0.428-1.171)	0.676 (0.403-1.135)	.14
Income					
Less than $25,000	30/67 (44.8%)	Ref[g]	Ref[h]	Ref[i]	Ref
$25,000 – 49,999	56/92 (60.9%)	**2.031 (1.068-3.864)**	**2.750 (1.393-5.429)**	**2.958 (1.461-5.988)**	**.003**
$50,000 – 100,000	61/138 (44.2%)	1.045 (0.579-1.885)	1.679 (0.866-3.254)	1.764 (0.886-3.515)	.11
More than $100,000	35/94 (37.2%)	0.732 (0.387-1.384)	1.164 (0.578-2.346)	1.141 (0.550-2.368)	.72
Health Insurance					
Yes	167/371 (45%)	0.452 (0.187-1.093)	0.557 (0.227-1.362)	0.429 (0.159-1.160)	.10
No	15/23 (65.2%)	Ref	Ref	Ref	Ref

	Agree[a]	Unadjusted OR (95% CI)	Age-adjusted[b] OR (95% CI)	Multivariably adjusted[c] OR (95% CI)	p-value for pairwise comparison vs. reference
Anxiety Severity (GAD-7)[d]					
Score 0-9	143/325 (44%)	Ref	Ref	Ref	Ref
Score 10-21	40/69 (58%)	**1.698 (1.003-2.874)**	1.231 (0.698-2.170)	1.241 (0.679-2.270)	.48
Psychological Openness[e]					
Score 0-16	25/48 (52.1%)	Ref	Ref	Ref	Ref
Score 17-32	158/346 (45.7%)	0.803 (0.438-1.470)	0.933 (0.501-1.738)	0.923 (0.488-1.747)	.81
Help-seeking Propensity[e]					
Score 0-16	13/28 (46.4%)	Ref	Ref	Ref	Ref
Score 17-32	170/366 (46.4%)	1.038 (0.480-2.244)	1.379 (0.622-3.061)	1.687 (0.731-3.897)	.22
Indifference to Anxiety Stigma[e]					
Score 0-16	23/34 (67.6%)	Ref	Ref	Ref	Ref
Score 17-32	159/358 (44.4%)	**0.396 (0.187-0.837)**	**0.453 (0.212-0.967)**	0.483 (0.224-1.043)	.064
Past Mental Health Service Use					
Yes	76/148 (51.4%)	1.393 (0.921-2.105)	1.080 (0.690-1.690)	1.122 (0.662-1.901)	.67
No	103/242 (42.6%)	Ref	Ref	Ref	Ref
Unmet Mental Health Need					
Yes	83/159 (52.2%)	1.463 (0.966-2.215)	0.971 (0.598-1.578)	0.967 (0.583-1.603)	.90
No	92/217 (42.4%)	Ref	Ref	Ref	Ref
Region					
Midwest	22/60 (36.7%)	0.584 (0.324-1.052)	0.567 (0.312-1.031)	0.586 (0.320-1.074)	.08
Northeast	28/69 (40.6%)	0.705 (0.407-1.224)	0.671 (0.383-1.176)	0.646 (0.362-1.152)	.14
West	114/229 (49.8%)	0.925 (0.445-1.920)	0.992 (0.470-2.096)	1.085 (0.506-2.329)	.83
South	16/34 (47.1%)	Ref[j]	Ref[k]	Ref[l]	Ref

[a]Agree indicates agreement with the use of text messaging to communicate with a professional to receive support for managing anxiety. No Agree indicates the respondent did not indicate agreement with the use of text messaging to communicate with a professional to receive support for managing anxiety.
[b]Adjusted for age only.
[c]Adjusted for age and history of anxiety. Statistically significant odds ratios are represented in bold.
[d]Score of ≥ 10 on the GAD-7 represents a reasonable cut-point for identifying cases of GAD.
[e]Higher scores indicate more positive attitudes toward seeking professional psychological help.
[f]Overall test of effect, degrees of freedom = 5, $P<.001$

[g]Overall test of effect, degrees of freedom = 3, P=.01
[h]Overall test of effect, degrees of freedom = 3, P=.01
[i]Overall test of effect, degrees of freedom = 3, P=.005
[j]Overall test of effect, degrees of freedom = 3, P=.26
[k]Overall test of effect, degrees of freedom = 3, P=.20
[l]Overall test of effect, degrees of freedom = 3, P=.20

Multivariable logistic regression models for attitudes toward using **voice call** to communicate with a professional to receive support for managing **anxiety.**

	Agree[a]	Unadjusted OR(95% CI)	Age-adjusted[b] OR (95% CI)	Multivariably adjusted[c] OR (95% CI)	p-value for pairwise comparison vs. reference
Age					
18-24	45/59 (76.3%)	Ref[f]	N/A	N/A	N/A
25-34	68/98 (69.4%)	0.705 (0.337-1.475)	N/A	N/A	N/A
35-44	40/46 (87%)	2.074 (0.728-5.908)	N/A	N/A	N/A
45-54	47/58 (81%)	1.329 (0.546-3.234)	N/A	N/A	N/A
55-64	36/55 (65.5%)	0.622 (0.273-1.419)	N/A	N/A	N/A
65+	51/79 (64.6%)	0.721 (0.330 – 1.575)	N/A	N/A	N/A
Age					
Less than 50	110/163 (67.5%)	Ref	N/A	N/A	N/A
50+	177/232 (76.3%)	0.743 (0.470-1.175)	N/A	N/A	N/A
Education					
Less than bachelor's degree	228/312 (73.1%)	Ref	Ref	Ref	Ref
Bachelor's degree or higher	59/83 (71.1%)	1.076 (0.619-1.870)	1.096 (0.629-1.910)	1.225 (0.695-2.159)	.48
Income					
Less than $25,000	48/67 (71.6%)	Ref[g]	Ref[h]	Ref[i]	Ref
$25,000 – 49,999	66/92 (71.7%)	1.076 (0.524-2.211)	1.130 (0.537-2.374)	1.316 (0.613-2.823)	.48
$50,000 – 100,000	97/138 (70.3%)	0.933 (0.484-1.799)	1.014 (0.490-2.099)	1.090 (0.516-2.303)	.82
More than $100,000	74/94 (78.7%)	1.460 (0.697-3.061)	1.582 (0.713-3.512)	1.820 (0.796-4.160)	.16
Health Insurance					
Yes	267/371 (72%)	0.579 (0.192-1.746)	0.598 (0.197-1.820)	0.476 (0.135-1.686)	.25
No	19/23 (82.6%)	Ref	Ref	Ref	Ref

	Agree[a]	Unadjusted OR(95% CI)	Age-adjusted[b] OR (95% CI)	Multivariably adjusted[c] OR (95% CI)	p-value for pairwise comparison vs. reference
Anxiety Severity (GAD-7)[d]					
Score 0-9	240/325 (73.8%)	Ref	Ref	Ref	Ref
Score 10-21	47/69 (68.1%)	0.703 (0.399-1.239)	0.612 (0.329-1.139)	0.674 (0.348-1.306)	.24
Psychological Openness[e]					
Score 0-16	38/48 (79.2%)	Ref	Ref	Ref	Ref
Score 17-32	249/346 (72%)	0.728 (0.348-1.522)	0.747 (0.355-1.570)	0.795 (0.375-1.683)	.55
Help-seeking Propensity[e]					
Score 0-16	18/28 (64.3%)	Ref	Ref	Ref	Ref
Score 17-32	269/366 (73.5%)	1.661 (0.740-3.730)	1.795 (0.784-4.108)	2.089 (0.893-4.889)	.09
Indifference to Anxiety Stigma[e]					
Score 0-16	11/34 (32.4%)	Ref	Ref	Ref	Ref
Score 17-32	258/358 (72.1%)	0.719 (0.303-1.707)	0.742 (0.311-1.772)	0.640 (0.254-1.616)	.35
Past Mental Health Service Use					
Yes	108/148 (73%)	0.941 (0.591-1.499)	0.889 (0.539-1.468)	0.971 (0.539-1.749)	.92
No	175/242 (72.3%)	Ref	Ref	Ref	Ref
Unmet Mental Health Need					
Yes	121/159 (76.1%)	1.358 (0.843-2.186)	1.356 (0.783-2.346)	1.476 (0.830-2.626)	.18
No	151/217 (69.6%)	Ref	Ref	Ref	Ref
Region					
Midwest	39/60 (65%)	0.601 (0.323-1.118)	0.599 (0.322-1.115)	0.584 (0.311-1.097)	.09
Northeast	49/69 (71%)	0.755 (0.413-1.382)	0.752 (0.410-1.376)	0.751 (0.403-1.401)	.37
West	172/229 (75.1%)	0.924 (0.392-2.179)	0.933 (0.396-2.202)	1.079 (0.437-2.662)	.87
South	24/34 (70.6%)	Ref[j]	Ref[k]	Ref[l]	Ref

[a]Agree indicates agreement with the use of voice call to communicate with a professional to receive support for managing anxiety. No Agree indicates the respondent did not indicate agreement with the use of voice call to communicate with a professional to receive support for managing anxiety.

[b]Adjusted for age only.

[c]Adjusted for age and history of anxiety. Statistically significant odds ratios are represented in bold.

[d]Score of ≥ 10 on the GAD-7 represents a reasonable cut-point for identifying cases of GAD.

[e]Higher scores indicate more positive attitudes toward seeking professional psychological help.

[f]Overall test of effect, degree of freedom = 5, P=.13

[g]Overall test of effect, degree of freedom = 3, P=.56

[h]Overall test of effect, degree of freedom = 3, P=.54

[i]Overall test of effect, degree of freedom = 3, P=.39

[j]Overall test of effect, degree of freedom = 3, P=.41

[k]Overall test of effect, degree of freedom = 3, P=.40
[l]Overall test of effect, degree of freedom = 3, P=.34

Multivariable logistic regression models for attitudes toward using **mobile apps** to communicate with a professional to receive support for managing **anxiety.**

	Agree[a]	Unadjusted OR (95% CI)	Age-adjusted[b] OR (95% CI)	Multivariably adjusted[c] OR (95% CI)	p-value for pairwise comparison vs. reference
Age					
18-24	37/59 (62.7%)	Ref[f]	N/A	N/A	N/A
25-34	54/98 (55.1%)	0.730 (0.377-1.413)	N/A	N/A	N/A
35-44	31/46 (67.4%)	1.229 (0.546-2.766)	N/A	N/A	N/A
45-54	24/58 (41.4%)	**0.420 (0.200-0.882)**	N/A	N/A	N/A
55-64	16/55 (29.1%)	**0.250 (0.114-0.550)**	N/A	N/A	N/A
65+	27/79 (34.2%)	**0.373 (0.183-0.763)**	N/A	N/A	N/A
Age					
Less than 50	54/163 (33.1%)	Ref	N/A	N/A	N/A
50+	135/232 (58.2%)	**0.392 (0.257-0.598)**	N/A	N/A	N/A
Education					
Less than bachelor's degree	142/312 (45.5%)	Ref	Ref	Ref	Ref
Bachelor's degree or higher	47/83 (56.6%)	0.612 (0.371-1.007)	0.663 (0.398-1.104)	0.671 (0.397-1.134)	.14
Income					
Less than $25,000	34/67 (50.7%)	Ref[g]	Ref[h]	Ref[i]	Ref
$25,000 – 49,999	48/92 (52.2%)	1.042 (0.550-1.974)	1.434 (0.730-2.815)	1.510 (0.749-3.044)	.25
$50,000 – 100,000	67/138 (48.6%)	0.926 (0.511-1.676)	1.550 (0.790-3.039)	1.498 (0.741-3.027)	.26
More than $100,000	40/94 (42.6%)	0.688 (0.364-1.301)	1.131 (0.559-2.290)	1.055 (0.505-2.204)	.89
Health Insurance					
Yes	175/371 (47.2%)	0.724 (0.309-1.693)	0.907 (0.382-2.151)	0.679 (0.264-1.747)	.42
No	13/23 (56.5%)	Ref	Ref	Ref	Ref
Anxiety Severity (GAD-7)[d]					

	Agree[a]	Unadjusted OR (95% CI)	Age-adjusted[b] OR (95% CI)	Multivariably adjusted[c] OR (95% CI)	p-value for pairwise comparison vs. reference
Score 0-9	146/325 (44.9%)	Ref	Ref	Ref	Ref
Score 10-21	43/69 (62.3%)	**1.914 (1.121-3.268)**	1.412 (0.795-2.508)	1.788 (0.960-3.331)	.07
Psychological Openness[e]					
Score 0-16	28/48 (58.3%)	Ref	Ref	Ref	Ref
Score 17-32	161/346 (46.5%)	0.657 (0.356-1.212)	0.754 (0.402-1.413)	0.791 (0.416-1.505)	.48
Help-seeking Propensity[e]					
Score 0-16	16/28 (57.1%)	Ref	Ref	Ref	Ref
Score 17-32	173/366 (47.3%)	0.709 (0.326-1.542)	0.922 (0.414-2.055)	1.058 (0.461-2.430)	.90
Indifference to Anxiety Stigma[e]					
Score 0-16	21/34 (61.8%)	Ref	Ref	Ref	Ref
Score 17-32	168/358 (46.9%)	0.578 (0.280-1.191)	0.663 (0.318-1.382)	0.709 (0.335-1.502)	.37
Past Mental Health Service Use					
Yes	76/148 (51.4%)	1.218 (0.805-1.841)	0.904 (0.575-1.419)	1.256 (0.734-2.150)	.41
No	109/242 (45%)	Ref	Ref	Ref	Ref
Unmet Mental Health Need					
Yes	91/159 (57.2%)	**1.903 (1.251-2.896)**	1.386 (0.852-2.253)	1.548 (0.926-2.587)	.10
No	89/217 (41%)	Ref	Ref	Ref	Ref
Region					
Midwest	22/60 (36.7%)	**0.521 (0.289-0.938)**	**0.502 (0.276-0.913)**	**0.495 (0.268-0.914)**	**.02**
Northeast	27/69 (39.1%)	0.606 (0.348-1.057)	**0.566 (0.321-0.998)**	**0.551 (0.307-0.991)**	.047
West	121/229 (52.8%)	1.001 (0.467-2.148)	1.020 (0.467-2.225)	1.187 (0.535-2.634)	.67
South	16/34 (47.1%)	Ref[j]	Ref[k]	Ref[l]	Ref

[a]Agree indicates agreement with the use of mobile apps to communicate with a professional to receive support for managing anxiety. No Agree indicates the respondent did not indicate agreement with the use of mobile apps to communicate with a professional to receive support for managing anxiety.
[b]Adjusted for age only.
[c]Adjusted for age and history of anxiety. Statistically significant odds ratios are represented in bold.
[d]Score of ≥ 10 on the GAD-7 represents a reasonable cut-point for identifying cases of GAD.
[e]Higher scores indicate more positive attitudes toward seeking professional psychological help.
[f]Overall test of effect, degree of freedom = 5, P<.001
[g]Overall test of effect, degree of freedom = 3, P= .51
[h]Overall test of effect, degree of freedom = 3, P=.48
[i]Overall test of effect, degree of freedom = 3, P=.42

[j]Overall test of effect, degree of freedom = 3, *P*=.08
[k]Overall test of effect, degree of freedom = 3, *P*=.05
[l]Overall test of effect, degree of freedom = 3, *P*=.04

Multivariable logistic regression models for attitudes toward using **video call** to communicate with a professional to receive support for managing **anxiety.**

	Agree[a]	Unadjusted OR (95% CI)	Age-adjusted[b] OR (95% CI)	Multivariably adjusted[c] OR (95% CI)	p-value for pairwise comparison vs. reference
Age					
18-24	40/59 (67.8%)	Ref[f]	N/A	N/A	N/A
25-34	72/98 (73.5%)	1.315 (0.649-2.666)	N/A	N/A	N/A
35-44	39/46 (84.8%)	**2.646 (1.001-6.997)**	N/A	N/A	N/A
45-54	41/58 (70.7%)	1.146 (0.522-2.514)	N/A	N/A	N/A
55-64	31/55 (56.4%)	0.669 (0.309-1.449)	N/A	N/A	N/A
65+	38/79 (48.1%)	0.531 (0.260-1.086)	N/A	N/A	N/A
Age					
Less than 50	89/163 (54.6%)	Ref	N/A	N/A	N/A
50+	172/232 (74.1%)	**0.478 (0.309-0.737)**	N/A	N/A	N/A
Education					
Less than bachelor's degree	210/312 (67.3%)	Ref	Ref	Ref	Ref
Bachelor's degree or higher	51/83 (61.4%)	1.300 (0.779-2.170)	1.417 (0.839-2.393)	1.361 (0.796-2.327)	.26
Income					
Less than $25,000	42/67 (62.7%)	Ref[g]	Ref[g]	Ref[h]	Ref
$25,000 – 49,999	64/92 (69.6%)	1.298 (0.658-2.559)	1.839 (0.891-3.797)	2.018 (0.959-4.246)	.064
$50,000 – 100,000	81/138 (58.7%)	0.853 (0.461-1.580)	1.447 (0.714-2.933)	1.458 (0.705-3.018)	.31
More than $100,000	73/94 (77.7%)	1.999 (0.983-4.063)	**3.380 (1.535-7.442)**	**3.343 (1.491-7.494)**	**.003**
Health Insurance					
Yes	244/371 (65.8%)	0.905 (0.362-2.259)	1.125 (0.443-2.854)	1.237 (0.470-3.259)	.67
No	16/23 (69.6%)	Ref	Ref	Ref	Ref
Anxiety Severity (GAD-7)[d]					

	Agree[a]	Unadjusted OR (95% CI)	Age-adjusted[b] OR (95% CI)	Multivariably adjusted[c] OR (95% CI)	p-value for pairwise comparison vs. reference
Score 0-9	202/313 (64.5%)	Ref	Ref	Ref	Ref
Score 10-21	56/78 (71.8%)	0.815 (0.472-1.405)	**0.522 (0.284-0.959)**	**0.509 (0.269-0.965)**	**.04**
Psychological Openness[e]					
Score 0-16	27/48 (56.3%)	Ref	Ref	Ref	Ref
Score 17-32	233/346 (67.3%)	1.743 (0.942-3.224)	**2.054 (1.087-3.883)**	**2.144 (1.118-4.110)**	**.02**
Help-seeking Propensity[e]					
Score 0-16	14/28 (50%)	Ref	Ref	Ref	Ref
Score 17-32	246/366 (67.2%)	**2.216 (1.022-4.805)**	**3.013 (1.342-6.765)**	**3.502 (1.502-8.161)**	**.004**
Indifference to Anxiety Stigma[e]					
Score 0-16	28/48 (58.3%)	Ref	Ref	Ref	Ref
Score 17-32	230/343 (67.1%)	1.154 (0.552-2.415)	1.355 (0.638-2.880)	1.386 (0.644-2.982)	.40
Past Mental Health Service Use					
Yes	97/148 (65.5%)	0.922 (0.594-1.431)	0.660 (0.404-1.078)	0.717 (0.406-1.266)	.25
No	161/242 (66.5%)	Ref	Ref	Ref	Ref
Unmet Mental Health Need					
Yes	117/159 (73.6%)	**1.874 (1.190-2.949)**	1.468 (0.870-2.477)	1.581 (0.918-2.723)	.10
No	128/217 (59%)	Ref	Ref	Ref	Ref
Region					
Midwest	35/60 (58.3%)	0.574 (0.319-1.033)	0.559 (0.308-1.015)	0.593 (0.324-1.085)	.09
Northeast	45/69 (65.2%)	0.878 (0.486-1.588)	0.834 (0.457-1.520)	0.910 (0.492-1.685)	.77
West	161/229 (70.3%)	0.708 (0.319-1.569)	0.714 (0.318-1.604)	0.750 (0.331-1.698)	.49
South	19/34 (55.9%)	Ref[j]	Ref[k]	Ref[l]	Ref

[a]Agree indicates agreement with the use of video call to communicate with a professional to receive support for managing anxiety. No Agree indicates the respondent did not indicate agreement with the use of video call to communicate with a professional to receive support for managing anxiety.
[b]Adjusted for age only.
[c]Adjusted for age and history of anxiety. Statistically significant odds ratios are represented in bold.
[d]Score of ≥ 10 on the GAD-7 represents a reasonable cut-point for identifying cases of GAD.
[e]Higher scores indicate more positive attitudes toward seeking professional psychological help.
[f]Overall test of effect, degree of freedom = 5, *P*=.01
[g]Overall test of effect, degree of freedom = 3, *P*=.04
[h]Overall test of effect, degree of freedom = 3, *P*=.01
[i]Overall test of effect, degree of freedom = 3, *P*=.01
[j]Overall test of effect, degree of freedom = 3, *P*=.29

[k]Overall test of effect, degree of freedom = 3, P=.27
[l]Overall test of effect, degree of freedom = 3, P=.38
