# Supplementary material for: Black American women’s attitudes toward seeking mental health services and use of mobile technology to support the management of anxiety
Source: JAMIA Open. 2023 Oct 17;6(4):ooad088. doi: 10.1093/jamiaopen/ooad088 (PMC10582519; doi:10.1093/jamiaopen/ooad088)
Supplement: ooad088_Supplementary_Data [file ooad088_supplementary_data.zip › Supplementary_File_1_Attitudes_Toward_Seeking_Mental_Health_Services_and_Use_of_Mobile_Technology_Survey.pdf]

Supplementary File 1: Attitudes toward seeking mental health services and use of mobile technology survey

Demographic Information

1. Do you consider yourself Hispanic or Latino?

- ○ Yes
- ○ No

2. Which of the following describe you? *Select all that apply*

- ☐ American Indian or Alaskan Native
- ☐ Asian
- ☐ Black or African American
- ☐ Native Hawaiian or Other Pacific Islander
- ☐ White
- ☐ Prefer Not to Answer

3. What is your age?

____

4. Which best describes your gender?

- ◯ Female
- ◯ Male
- ◯ Gender Variant/ Non-Conforming
- ◯ Not Listed, please specify ____

5. What is the highest level of education that you have completed?

- ◯ Less than a high school diploma
- ◯ High school diploma or GED
- ◯ Some college, less than 4-year degree
- ◯ Bachelor's degree or higher
- ◯

6. Which category best describes your current annual household income (i.e., per year)? Be sure to include income from all sources, such as salary and wages, child support, interest, public assistance, and pensions.

- ◯ Less than $10,000
- ◯ $10,000 – 24,999
- ◯ $25,000 – 49,999
- ◯ $50,000 – 100,000
- ◯ More than $100,000

Your Feelings

7. Over the last 2 weeks, how often have you been bothered by any of the following problems?

	Not at all	Several days	More than half the days	Nearly every day
a. Little interest or pleasure in doing things.	○	○	○	○
b. Feeling down, depressed, or hopeless.	○	○	○	○
c. Trouble falling or staying asleep, sleeping too much.	○	○	○	○
d. Feeling tired or having little energy.	○	○	○	○
e. Poor appetite or overeating.	○	○	○	○
f. Feeling bad about yourself or that you are a failure or have let yourself or your family down.	○	○	○	○
g. Trouble concentrating on things, such as reading the newspaper or watching television.	○	○	○	○

h. Moving or speaking so slowly that other people could have noticed. Or the opposite; being so fidgety or restless that you have been moving around a lot more than usual.	○	○	○	○
i. Thoughts that you would be better off dead or of hurting yourself in some way.	○	○	○	○

8. If you selected <u>any</u> problems, how <u>difficult</u> have these problems made it for you to do your work, take care of things at home, or get along with other people?

○ Not difficult at all

○ Somewhat difficult

○ Very difficult

○ Extremely difficult

9. Over the last 2 weeks, how often have you been bothered by any of the following problems?

	Not at all	Several days	More than half the days	Nearly every day
a. Feeling nervous, anxious or on edge.	○	○	○	○
b. Not being able to stop or control worrying.	○	○	○	○
c. Worrying too much about different things.	○	○	○	○
d. Trouble relaxing.	○	○	○	○
e. Being so restless that it is hard to sit still.	○	○	○	○
f. Becoming easily annoyed or irritable.	○	○	○	○
g. Feeling afraid as if something awful might happen.	○	○	○	○

10. If you selected <u>any</u> problems, how <u>difficult</u> have these problems made it for you to do your work, take care of things at home, or get along with other people?

- ◯ Not difficult at all
- ◯ Somewhat difficult
- ◯ Very difficult
- ◯ Extremely difficult

Attitudes Toward Seeking Mental Health Services

<u>Definition of terms</u>
The term **professional** refers to individuals who have been trained to deal with mental health problems (e.g., psychologists, psychiatrists, social workers, and family physicians). The term **psychological problems** refers to reasons one might visit a professional. Similar terms include mental health concerns, emotional problems, mental troubles, and personal difficulties.

The term **anxiety** is defined as an emotion characterized by feelings of tension, worried thoughts and physical changes like increased blood pressure. The term **depression** is defined as a mood disorder that causes a persistent feeling of sadness and loss of interest.

Instructions: For each item, indicate whether you *Disagree*, *Somewhat Disagree*, are *Undecided*, *Somewhat Agree*, or *Agree*:

11. There are certain problems which should not be discussed outside of one's immediate family.

- ◯ Disagree
- ◯ Somewhat Disagree
- ◯ Undecided
- ◯ Somewhat Agree
- ◯ Agree

12. I would have a very good idea of what to do and who to talk to if I decided to seek professional help for psychological problems.

- ◯ Disagree
- ◯ Somewhat Disagree
- ◯ Undecided
- ◯ Somewhat Agree
- ◯ Agree

13. I would not want my significant other (spouse, partner, etc.) to know if I were suffering from **anxiety**.

- ◯ Disagree
- ◯ Somewhat Disagree
- ◯ Undecided
- ◯ Somewhat Agree
- ◯ Agree

14. I would not want my significant other (spouse, partner, etc.) to know if I were suffering from **depression**.

- ◯ Disagree
- ◯ Somewhat Disagree
- ◯ Undecided
- ◯ Somewhat Agree
- ◯ Agree

15. Keeping one's mind on a job is a good solution for avoiding personal worries and concerns.

- ◯ Disagree
- ◯ Somewhat Disagree
- ◯ Undecided
- ◯ Somewhat Agree
- ◯ Agree

16. If good friends asked my advice about a psychological problem, I might recommend that they see a professional.

- ◯ Disagree
- ◯ Somewhat Disagree
- ◯ Undecided
- ◯ Somewhat Agree
- ◯ Agree

17. Having **anxiety** carries with it a burden of shame.

- ◯ Disagree
- ◯ Somewhat Disagree
- ◯ Undecided
- ◯ Somewhat Agree
- ◯ Agree

18. Having **depression** carries with it a burden of shame.

- ◯ Disagree
- ◯ Somewhat Disagree
- ◯ Undecided
- ◯ Somewhat Agree
- ◯ Agree

19. It is probably best not to know *everything* about oneself.

- ◯ Disagree
- ◯ Somewhat Disagree
- ◯ Undecided
- ◯ Somewhat Agree
- ◯ Agree

20. If I were experiencing a serious psychological problem at this point in my life, I would be confident that I could find relief in psychotherapy.

- Disagree
- Somewhat Disagree
- Undecided
- Somewhat Agree
- Agree

21. People should work out their own problems; getting professional help should be a last resort.

- Disagree
- Somewhat Disagree
- Undecided
- Somewhat Agree
- Agree

22. If I were to experience psychological problems, I could get professional help if I wanted to.

- Disagree
- Somewhat Disagree
- Undecided
- Somewhat Agree
- Agree

23. Important people in my life would think less of me if they were to find out that I was dealing with **anxiety**.

- ○ Disagree
- ○ Somewhat Disagree
- ○ Undecided
- ○ Somewhat Agree
- ○ Agree

24. Important people in my life would think less of me if they were to find out that I was dealing with **depression**.

- ○ Disagree
- ○ Somewhat Disagree
- ○ Undecided
- ○ Somewhat Agree
- ○ Agree

25. Psychological problems, like many things, tend to work out by themselves.

- ○ Disagree
- ○ Somewhat Disagree
- ○ Undecided
- ○ Somewhat Agree
- ○ Agree

26. It would be relatively easy for me to find the time to see a professional for psychological problems.

- Disagree
- Somewhat Disagree
- Undecided
- Somewhat Agree
- Agree

27. There are experiences in my life I would not discuss with anyone.

- Disagree
- Somewhat Disagree
- Undecided
- Somewhat Agree
- Agree

28. I would want to get professional help if I were worried or upset for a long period of time.

- Disagree
- Somewhat Disagree
- Undecided
- Somewhat Agree
- Agree

29. I would be uncomfortable seeking professional help for **anxiety** because people in my social or business circles might find out about it.

- Disagree
- Somewhat Disagree
- Undecided
- Somewhat Agree
- Agree

30. I would be uncomfortable seeking professional help for **depression** because people in my social or business circles might find out about it.

- Disagree
- Somewhat Disagree
- Undecided
- Somewhat Agree
- Agree

31. Having been diagnosed with **anxiety** is a blot on a person’s life.

- ◯ Disagree
- ◯ Somewhat Disagree
- ◯ Undecided
- ◯ Somewhat Agree
- ◯ Agree

32. Having been diagnosed with **depression** is a blot on a person’s life.

- ◯ Disagree
- ◯ Somewhat Disagree
- ◯ Undecided
- ◯ Somewhat Agree
- ◯ Agree

33. There is something admirable in the attitude of people who are willing to cope with their conflicts and fears *without* resorting to professional help.

- ○ Disagree
- ○ Somewhat Disagree
- ○ Undecided
- ○ Somewhat Agree
- ○ Agree

34. If I believed I were having a mental breakdown, my first inclination would be to get professional attention.

- ○ Disagree
- ○ Somewhat Disagree
- ○ Undecided
- ○ Somewhat Agree
- ○ Agree

35. I would feel uneasy going to a professional because of what some people would think.

- ○ Disagree
- ○ Somewhat Disagree
- ○ Undecided
- ○ Somewhat Agree
- ○ Agree

36. People with strong characters can get over psychological problems by themselves and would have little need for professional help.

- ○ Disagree
- ○ Somewhat Disagree
- ○ Undecided
- ○ Somewhat Agree
- ○ Agree

37. I would willingly confide intimate matters to an appropriate person if I thought it might help me or a member of my family.

- ○ Disagree
- ○ Somewhat Disagree
- ○ Undecided
- ○ Somewhat Agree
- ○ Agree

38. Had I received treatment for **anxiety**, I would not feel that it ought to be “covered up.”

- ○ Disagree
- ○ Somewhat Disagree
- ○ Undecided
- ○ Somewhat Agree
- ○ Agree

39. Had I received treatment for **depression**, I would not feel that it ought to be "covered up."

- ○ Disagree
- ○ Somewhat Disagree
- ○ Undecided
- ○ Somewhat Agree
- ○ Agree

40. I would be embarrassed if my neighbor saw me going into the office of a professional who deals with psychological problems.

- ○ Disagree
- ○ Somewhat Disagree
- ○ Undecided
- ○ Somewhat Agree
- ○ Agree

Mental Health Service Use

These next questions are about treatment and counseling for problems with emotions, nerves or mental health. Please do not include treatment for alcohol or drug use. The answers that you give us about mental health treatment are important to this study's success, and will be used to help researchers and clinicians better understand the mental health service needs of Black women. We know that this information is personal, but remember your answers will be kept confidential.

41. The list below includes some of the places where people can get treatment or counseling for problems with their emotions, nerves, or mental health. During the <u>past 12 months</u>, did you receive any treatment or counseling for any problem you were having with your emotions, nerves, or mental health at any of the places listed below?

- An outpatient mental health clinic or center
- The office of a private therapist, psychologist, psychiatrist, social worker, or counselor that was not part of a clinic
- A doctor's office that was not part of a clinic
- An outpatient medical clinic
- A partial day hospital or day treatment program
- Some other place

○ Yes

○ No

○ Don't Know

○ Refused

42. Where did you receive mental health treatment or counseling during the past 12 months?
Select all that apply

☐ An outpatient mental health clinic or center

☐ The office of a private therapist, psychologist, psychiatrist, social worker, or counselor that was not part of a clinic

☐ A doctor's office that was not part of a clinic

☐ An outpatient medical clinic

☐ A partial day hospital or day treatment program

☐ Some other place, please specify

____

43. During the past 12 months, was there any time when you needed mental health treatment or counseling for yourself but didn’t get it?

- ◯ Yes
- ◯ No
- ◯ Don't Know
- ◯ Refused

44. Which of these statements explain why you did not get the mental health treatment or counseling you needed? *Select all that apply*

- ☐ You couldn’t afford the cost.
- ☐ You were concerned that getting mental health treatment or counseling might cause your neighbors or community to have a negative opinion of you.
- ☐ You were concerned that getting mental health treatment or counseling might have a negative effect on your job.
- ☐ Your health insurance does not cover any mental health treatment or counseling.
- ☐ Your health insurance does not pay enough for mental health treatment or counseling.
- ☐ You did not know where to go to get services.
- ☐ You were concerned that the information you gave the counselor might not be kept confidential.
- ☐ You were concerned that you might be committed to a psychiatric hospital or might have to take medicine.
- ☐ Some other reason or reasons, please specify ______________________________

45. Earlier, we asked did you receive any treatment or counseling for any problem you were having with your emotions, nerves, or mental health. The response options below contain possible sources of treatment, counseling or support that were not mentioned before. During the <u>past 12 months</u>, did you receive any treatment or counseling for any problem you were having with your emotions, nerves, or mental health at any of the places listed below?

- Spiritual or religious advisor, such as a pastor, priest, rabbi
- Acupuncturist or acupressurist
- Chiropractor
- Herbalist
- In-person support group or self-help group
- Online counseling (e.g., video call with therapist)
- Internet support group or chat room
- Telephone hotline Massage therapist
- Some other place

○ Yes

○ No

○ Don't Know

○ Refused

46. Where did you receive mental health treatment or counseling during the past 12 months?
Select all that apply

☐ Spiritual or religious advisor, such as a pastor, priest, rabbi

☐ Acupuncturist or acupressurist

☐ Chiropractor

☐ Herbalist

☐ In-person support group or self-help group

☐ Online counseling (e.g., video call with therapist)

☐ Internet support group or chat room

☐ Telephone hotline

- [ ] Massage therapist
- [ ] Some other place, please specify ______________________________

Mobile Phone Use

47. Do you currently own a mobile phone?

- ○ Yes
- ○ No

48. On average, how often do you use your phone to send text messages?

- ◯ Never
- ◯ Less than 1 time per week
- ◯ 1-6 times per week
- ◯ 1-3 times per day
- ◯ 4 or more times per day

49. On average, how often do you use your phone to access mobile applications (e.g., social media apps, news apps, navigation apps, etc.)?

- ○ Never
- ○ Less than 1 time per week
- ○ 1-6 times per week
- ○ 1-3 times per day
- ○ 4 or more times per day

50. Are you able to complete video calls on your phone (e.g., FaceTime, Skype, WhatsApp, etc.)?

- ○ Yes
- ○ No

51. On average, how often do you use your phone to complete video calls?

- ○ Never
- ○ Less than 1 time per week
- ○ 1-6 times per week
- ○ 1-3 times per day
- ○ 4 or more times per day

Use of Mobile Phone to Receive Mental Health Services

<u>Definition of terms</u>
The term professional refers to individuals who have been trained to deal with mental health problems (e.g., psychologists, psychiatrists, social workers, and family physicians). The term anxiety is defined as an emotion characterized by feelings of tension, worried thoughts and physical changes like increased blood pressure. The term depression is defined as a mood disorder that causes a persistent feeling of sadness and loss of interest.

Instructions: For the following statements, please read each sentence replacing the blank space with the listed options for each statement (e.g., text messaging, voice call, mobile app, or video call). For example, "I would feel comfortable communicating with a professional through <u>text messaging</u> to receive help for managing anxiety." Then select your response for each statement.

52. I would feel comfortable communicating with a professional through _________ to receive help for managing anxiety.

	Disagree	Somewhat Disagree	Undecided	Somewhat Agree	Agree
a. text messaging	○	○	○	○	○
b. voice call	○	○	○	○	○
c. mobile app	○	○	○	○	○
d. video call	○	○	○	○	○

53. I would feel comfortable communicating with a professional through _________ to receive help for managing **depression**.

	Disagree	Somewhat Disagree	Undecided	Somewhat Agree	Agree
a. text messaging	○	○	○	○	○
b. voice call	○	○	○	○	○
c. mobile app	○	○	○	○	○
d. video call	○	○	○	○	○

54. Having the option to use __________ to communicate with a professional if I am dealing with anxiety would be helpful for me.

	Disagree	Somewhat Disagree	Undecided	Somewhat Agree	Agree
a. text messaging	○	○	○	○	○
b. voice call	○	○	○	○	○
c. mobile app	○	○	○	○	○
d. video call	○	○	○	○	○

55. Having the option to use __________ to communicate with a professional if I am dealing with **depression** would be helpful for me.

	Disagree	Somewhat Disagree	Undecided	Somewhat Agree	Agree
a. text messaging	○	○	○	○	○
b. voice call	○	○	○	○	○
c. mobile app	○	○	○	○	○
d. video call	○	○	○	○	○

56. Would having the option to use a _________ to complete an appointment with a professional increase your access to mental health services?

	Yes	No
a. voice call	○	○
b. mobile app	○	○
c. video call	○	○

57. Would having the option to use a _________ to complete an appointment save you time traveling to a professional's office?

	Yes	No
a. voice call	○	○
b. mobile app	○	○
c. video call	○	○

58. Would having the option to use a _________ to complete an appointment with a professional be more convenient for you than an in-person appointment?

	Yes	No
a. voice call	○	○
b. mobile app	○	○
c. video call	○	○

59. Do you have any concerns about using a voice call to receive mental health treatment or counseling?

- ○ Yes
- ○ No

60. What are your concerns about using a voice call to receive mental health treatment or counseling?

____

____

____

____

____

61. Do you have any concerns about using a mobile app to receive mental health treatment or counseling?

- ◯ Yes
- ◯ No

62. What are your concerns about using a mobile app to receive mental health treatment or counseling?

____

____

____

____

____

63. Do you have any concerns about using a video call to receive mental health treatment or counseling?

- ◯ Yes
- ◯ No

64. What are your concerns about using a video call to receive mental health treatment or counseling?

____

____

____

____

____

65. Do you have any concerns about using text messaging to communicate with a professional about your mental health?

- ◯ Yes
- ◯ No

66. What are your concerns about using text messaging to communicate with a professional about your mental health?

____

____

____

____

____

67. Have you ever been diagnosed with **anxiety**?

- ◯ Yes
- ◯ No
- ◯ Don't Know
- ◯ Refused

68. Have you ever been diagnosed with **depression**?

- ◯ Yes
- ◯ No
- ◯ Don't Know
- ◯ Refused

69. Do you currently have health insurance?

- ◯ Yes
- ◯ No
- ◯ Don't Know

70. Does your health insurance pay for any type of mental health treatment or counseling services?

- ◯ Yes
- ◯ No
- ◯ Don't Know

71. Comments (Optional)

Thank you for completing the survey, and contributing to this important research!
